# Inappropriate Testing for Acute Viral Hepatitis Is Common—Impact of an Intervention Using the Electronic Health Record in a Tertiary Teaching Hospital in the United States

**DOI:** 10.31486/toj.19.0062

**Published:** 2020

**Authors:** Muhammad Bader Hammami, Kamran Hussaini, Anuj Chhaparia, Hamza Khalid, Robin Chamberland, Brent Neuschwander-Tetri

**Affiliations:** ^1^ Department of Medicine, University of California, Riverside, Riverside, CA; ^2^ Department of Medicine, VA Loma Linda Healthcare System, Loma Linda, CA; ^3^ Department of Medicine, Saint Louis University, Saint Louis, MO; ^4^ Department of Pathology, Saint Louis University, Saint Louis, MO

**Keywords:** *Cost control*, *health care costs*, *health expenditures*, *hepatitis*, *unnecessary procedures*

## Abstract

**Background:** Unnecessary laboratory tests contribute to the financial burden placed on hospitals, patients, insurers, and taxpayers. In our institution, we noted acute viral hepatitis serologic testing in patients with chronic liver disease, sometimes done repetitively, in the absence of substantially elevated aminotransferase levels. The goal of this study was to determine the frequency of unnecessary testing for acute hepatitis A and B infections and then reduce testing rates by implementing an intervention in the electronic health record.

**Methods:** In a 2-year period, 2 successive interventions questioning the appropriateness of ordering viral hepatitis serology based on transaminase elevation and prior serology results were implemented in the electronic health record system at Saint Louis University Hospital. The first intervention allowed providers to override the warning without providing a reason; the second intervention required justification to proceed with the order. Preintervention and postintervention appropriate and inappropriate testing proportions were compared using Fisher exact test.

**Results:** The electronic reminders resulted in a statistically significant reduction of inappropriate testing rates; however, testing rates remained high whether the provider had to justify overriding the automatic alert or not.

**Conclusion:** Our research demonstrated that the rates of inappropriate testing for acute viral hepatitis at our institution were unnecessarily high and showed that a simple intervention in the medical record system may be useful in reducing inappropriate testing. Our interventions were feasible and implemented at minimal cost. Similar interventions could be used to target other unnecessary tests, but education and additional interventions will likely be required to reduce unnecessary testing further.

## INTRODUCTION

As a percentage of gross domestic product (GDP), more money is spent on healthcare in the United States than in any other developed country.^1^ The Centers for Disease Control and Prevention reported that between the years 1975 and 2015, national health expenditures in the United States as a percent of GDP increased from 7.9% to 17.8%.^2^ In 1975, the United States spent $113 billion on personal healthcare, an average of $514 per person per year. In contrast, by 2015, spending had increased to $2.7 trillion, an average of $8,468 per person per year.^2^ A significant proportion of these costs has been attributed to unnecessary services or expenditures that providers can directly avoid by optimizing their practice.^3^ Laboratory tests, which play a vital role in the medical decision-making process, can contribute to excess healthcare expenditures when inappropriately used. A large proportion of unnecessary testing may be related to defensive medicine; poor understanding of disease processes; convenience of ordering and availability of tests, especially in panels or order sets; minimal restrictions on ordering tests; and the frequent lack of disincentives for ordering unnecessary tests.

The goal of the present research was to determine the rates of unnecessary acute viral hepatitis testing at a tertiary care university hospital and assess the impact of an electronic health record (EHR) educational guidance statement designed to reduce the frequency of inappropriate testing. This research was based on the anecdotal observation that serologic testing for acute hepatitis A and B is often ordered to evaluate patients with chronic liver disease who lack clinical evidence of acute liver injury or who have documented prior immunity to hepatitis A or B. We also aimed to identify the magnitude of a new and growing problem of inappropriate screening for hepatitis C with antibody testing in patients with a known history of hepatitis C, many of whom had been cured of their infection.

## METHODS

The study was conducted in accordance with the Declaration of Helsinki after approval by the institutional review board of Saint Louis University. A retrospective review of all hepatitis A virus immunoglobulin M (IgM) antibody (anti-HAV IgM), hepatitis A virus total antibody (anti-HAV total), hepatitis B virus core IgM antibody (anti-HBc IgM), hepatitis B virus core total antibody (anti-HBc total), hepatitis B virus surface antibody (anti-HBs), hepatitis C virus antibody (anti-HCV), and hepatitis C virus ribonucleic acid (HCV RNA) testing results with contemporaneous alanine aminotransferase (ALT) and aspartate aminotransferase (AST) values was performed at Saint Louis University Hospital. Data were collected from the hospital EHR systems, Epic and Meditech. The medical records service provided results and dates of the tests to the investigators in a deidentified manner for 3 time frames: preintervention, post first intervention, and post second intervention.

For this analysis, an ALT or AST level ≥250 U/L within 30 days of testing was chosen to be appropriate for ordering the acute viral hepatitis serologies tests: anti-HAV IgM and anti-HBc IgM. Thus, inappropriate serologies tests were anti-HAV IgM or anti-HBc IgM ordered with a contemporaneous ALT or AST level <250 U/L. Serologic tests without contemporaneous ALT and AST values available in the hospital EHR were excluded because these liver enzyme results were likely available to the providers from sources outside of the hospital record system.

In an additional analysis, other serologic testing considered to be inappropriate was anti-HCV testing in patients with documented prior positive anti-HCV or HCV RNA tests, anti-HAV (IgM or total antibody) testing with documented HAV immunity defined as a prior positive anti-HAV total test (>6 months prior), and anti-HBc total testing in patients with a documented prior positive anti-HBc total test (>6 months prior).

### Interventions

Two interventions to reduce inappropriate ordering were implemented sequentially as shown in Table 1.

**Table 1.** Inappropriate Viral Hepatitis Testing Criteria

Data Collection Period	Test	Inappropriate Test Criteria
First and second intervention	Anti-HAV IgM	ALT and AST <250 U/L or prior positive anti-HAV total antibody positive (>6 months prior)
	Anti-HBc IgM	ALT and AST <250 U/L or prior positive anti-HBs antibody positive (>6 months prior)
Second intervention only	Anti-HAV total	Prior anti-HAV total antibody positive
	Anti-HBc total	Prior anti-HBc total antibody positive
	Anti-HCV	Prior anti-HCV or HCV RNA positive

ALT, alanine aminotransferase; anti-HAV IgM, hepatitis A virus immunoglobulin M antibody; anti-HAV total, hepatitis A virus total antibody; anti-HBc IgM, hepatitis B virus core immunoglobulin M antibody; anti-HBc total, hepatitis B virus core total antibody; anti-HBs, hepatitis B virus surface antibody; anti-HCV, hepatitis C virus antibody; AST, aspartate aminotransferase; HCV RNA, hepatitis C virus RNA.

#### First Intervention

In the first intervention, a 1-step warning was implemented in the Epic EHR system in December 2015. An advisory screen was programmed to appear when an anti-HAV IgM or anti-HBc IgM test was ordered for a patient whose highest ALT or AST value within the prior 30 days was <250 U/L or for a patient whose previous serology demonstrated immunity to HAV (anti-HAV total positive) or HBV (anti-HBs positive). The warning was automatically generated and read as follows: “Selected laboratory test is valuable for diagnosing acute hepatitis for patients with current ALT or AST greater than 250 U/L and without known immunity to HAV (anti-HAV total positive) or HBV (anti-HBs positive).” The advisory screen also showed the most recent and highest ALT and AST values within the prior 30 days, as well as prior results of anti-HAV total and anti-HBs when available in the hospital EHR system. The provider could override the warning without justifying why the testing was needed.

#### Second Intervention

Because the initial 1-step intervention was easy to override, a modified 2-step warning was implemented in January 2017. Similar to the first intervention, an advisory screen was programmed to trigger when anti-HAV IgM or anti-HBc IgM tests were ordered for patients whose highest ALT or AST values were <250 U/L within the prior 30 days or for patients with previous serologic evidence of immunity to HAV (anti-HAV total positive) or HBV (anti-HBs positive). The warning was automatically generated and read as follows: “This is an inappropriate test based on available results. The selected test should be used for diagnosing acute viral hepatitis in patients with current ALT or AST greater than 250 U/L. Ordering this test is of no clinical or diagnostic value given the available results and adds unnecessary health care costs.” The advisory screen also showed the most recent and highest ALT and AST values within the prior 30 days, as well as prior results of anti-HAV total and anti-HBs tests if available. Unlike the first intervention, the second intervention allowed the provider to override only after providing a justification for the test.

An advisory screen was also programmed to trigger when an anti-HCV test was ordered for a patient with a prior positive anti-HCV or HCV RNA test, when HAV total antibody testing was ordered for a patient with a prior positive anti-HAV total test, or when anti-HBc total testing was ordered for a patient with a prior positive anti-HBc total test. The provider could override the warning only after providing a justification for the test.

### Analysis

The numbers and proportions of preintervention and postintervention appropriate and inappropriate tests were compared using Fisher exact test. *P* values ≤0.05 were regarded as statistically significant.

## RESULTS

Tables 2 and 3 present the preintervention and postintervention results.

**Table 2.** Numbers and Rates of Inappropriate Acute Viral Hepatitis Serology Testing Before and After Interventions

		Test	
Data Collection Period	Variable	Anti-HAV IgM Test	Anti-HBc IgM
Preintervention	Total eligible tests analyzed, n	2,439	4,462
	ALT or AST ≥250 U/L, n (%)	228 (9.3)	280 (6.3)
	ALT or AST <250 U/L, n (%)	2,211 (90.7)	4,182 (93.7)
	Rate of inappropriate testing, %	90.7	93.7
Post first intervention	Total tests ordered, n	1,495	1,830
	Total eligible tests analyzed, n	1,311	1,573
	ALT or AST ≥250 U/L, n (%)	255 (19.5)	266 (16.9)
	ALT or AST <250 U/L, n (%) or serologic evidence of immunity	1,056 (80.5)	1,307 (83.1)
	Rate of inappropriate testing, %	80.5	83.1
	*P* value	<0.0001	<0.0001
Post second intervention	Total tests ordered, n	1,159	1,437
	Total eligible tests analyzed, n	998	1,184
	ALT or AST ≥250 U/L, n (%)	199 (19.9)	200 (16.9)
	ALT or AST <250 U/L, n (%) or serologic evidence of immunity	799 (80.1)	984 (83.1)
	Rate of inappropriate testing, %	80.1	83.1
	*P* value	<0.0001	<0.0001

Notes: Serologic evidence of immunity to HAV and HBV is defined as the presence a positive anti-HAV total (>6 months) and positive anti-HBs (>6 months), respectively. Eligible tests are less than the total tests, as tests without AST or ALT values within 30 days of ordering the serology were excluded. *P* value is 2-sided comparing the preintervention and postintervention appropriate and inappropriate proportions using Fisher exact test.

ALT, alanine aminotransferase; anti-HAV IgM, hepatitis A virus immunoglobulin M antibody; anti-HAV total, hepatitis A virus total antibody; anti-HBc IgM, hepatitis B virus core immunoglobulin M antibody; anti-HBs, hepatitis B virus surface antibody; HAV, hepatitis A virus; HBV, hepatitis B virus; AST, aspartate aminotransferase.

**Table 3.** Numbers and Rates of Inappropriate Chronic Viral Hepatitis Serology Testing Preintervention and After the Second Intervention

		Test		
Data Collection Period	Variable	Anti-HCV	Anti-HAV Total	Anti-HBc Total
Preintervention	Total tests ordered, n	10,263	4,398	4,575
	Total positive tests, n (%)	1,537 (15.0)	2,023 (46.0)	784 (17.1)
	Number of tests ordered inappropriately, n (%)	361 (3.5)	222 (5.0)	53 (1.2)
Post second intervention	Total tests ordered, n	4,945	993	2,273
	Total positive tests, n (%)	571 (11.5)	516 (52.0)	296 (13.0)
	Number of tests ordered inappropriately, n (%)	158 (3.2)	13 (1.3)	29 (1.3)
	*P* value	0.34	<0.0001	0.72

Note: *P* value is 2-sided comparing the rates of inappropriate testing after the intervention to the rates before any intervention.

Anti-HAV total, hepatitis A virus total antibody; anti-HBc total, hepatitis B virus core total antibody; anti-HCV, hepatitis C virus antibody.

### Preintervention Results

Preintervention data were collected for the period January 2010 through November 2014. Because of an error in data collection, no data were collected from June 2013 through December 2013. During the 52 months of data collection, anti-HAV IgM and anti-HBc IgM were tested a total of 2,439 and 4,462 times, respectively. Of these, 2,211 (90.7%) anti-HAV IgM and 4,182 (93.7%) anti-HBc IgM tests were judged retrospectively to be inappropriate because they were obtained for patients who did not have biochemical evidence of acute liver injury or who had known immunity to the virus being tested.

During the preintervention period, anti-HCV was tested 10,263 times, was positive 1,537 times (15.0%), and was indeterminate 45 times (0.4%). The 45 indeterminate anti-HCV tests were appropriately followed by HCV RNA testing only 11 times (24.4%). Inappropriate anti-HCV testing in patients with a known positive anti-HCV test was performed 179 times, comprising 11.6% of the positive anti-HCV results. Inappropriate anti-HCV testing following a positive HCV RNA test was performed 182 times, accounting for 11.8% of the positive anti-HCV results. These 2 tests brought the total number of inappropriate anti-HCV tests to 361, or 3.5% of the total anti-HCV tests.

Anti-HAV total testing was performed 4,398 times and was positive 2,023 times (46.0%). Anti-HAV total testing in patients with a known positive anti-HAV total test was performed 222 times, so 5.0% of all anti-HAV total tests were inappropriate.

Anti-HBc total testing was performed 4,575 times and was positive 784 times (17.1%). Anti-HBc total testing in patients with a known positive anti-HBc total result was performed 53 times, so 1.2% of all anti-HBc total tests were inappropriate.

### Post First Intervention Results

After the first 1-step warning was implemented, the results and number of anti-HAV IgM and anti-HBc IgM tests were collected during the subsequent 12 months (December 2015 through November 2016), and we compared the proportions of appropriate and inappropriate tests to the preintervention proportions.

Anti-HAV IgM was tested 1,495 times during the first intervention period. The test was ordered 184 times without available ALT or AST results within 30 days of testing, and these tests were excluded from the analysis, so the number of tests eligible for analysis was 1,311. The anti-HAV IgM test was appropriately ordered 255 times (19.5%) and inappropriately ordered 1,056 times (80.5%). Of the 1,495 anti-HAV IgM tests performed, 1,472 tests (98.5%) were nonreactive (negative), 12 tests (0.8%) were reactive (positive), and 11 tests (0.7%) were indeterminate. Of the 12 reactive tests, 2 were performed appropriately, 6 were inappropriate according to the criteria defined for this study, and 4 were done without ALT or AST results within the prior 30 days. Of the 11 indeterminate tests, 1 was performed appropriately, 5 were inappropriate, and 5 were done without ALT or AST results within the prior 30 days.

Anti-HBc IgM was tested 1,830 times during the first intervention period. The test was ordered 257 times without ALT or AST results within 30 days of testing, and these tests were excluded from the analysis, so the number of tests eligible for analysis was 1,573. The anti-HBc IgM test was appropriately ordered 266 times (16.9%) and inappropriately 1,307 times (83.1%). Of the 1,830 anti-HBc IgM antibody tests, 1,807 tests (98.7%) were nonreactive (negative), 16 tests (0.9%) were reactive (positive), and 7 tests (0.4%) were indeterminate. Of the 16 reactive tests, 2 were performed appropriately, 12 were inappropriate, and 2 were done without ALT or AST results within the prior 30 days. Of the 7 indeterminate tests, 2 were performed appropriately, 3 were inappropriate, and 2 were done without ALT or AST results within the prior 30 days.

Based on these data, the 1-step advisory screen achieved statistically significant (*P*<0.0001) but relatively small 10.2 and 10.6 percentage point reductions of inappropriate anti-HAV IgM and anti-HBc IgM testing, respectively.

### Post Second Intervention Results

As stated previously, the second intervention was a modification of the electronic warning that required the provider to justify ordering an inappropriate test, thus creating a 2-step advisory screen. After this warning was implemented, data on anti-HAV IgM and anti-HBc IgM testing and the highest values of ALT or AST within 30 days before testing were collected for an 11-month period (January 2017 through November 2017). Serologies without available contemporaneous ALT or AST values were excluded. We compared the rates of inappropriate testing after the second intervention to the rates of preintervention inappropriate testing.

After implementing the second intervention, anti-HAV IgM was tested 1,159 times. Tests were ordered 161 times without ALT or AST results within 30 days of testing, so 998 tests were eligible for analysis. Of these, the anti-HAV IgM test was appropriately ordered 199 times (19.9%) and inappropriately ordered 799 times (80.1%). All the 1,159 anti-HAV IgM tests obtained during this period were nonreactive.

Anti-HBc IgM was tested 1,437 times after the second intervention. The test was ordered 253 times without ALT or AST results within 30 days of testing, so 1,184 tests were eligible for analysis. The anti-HBc IgM test was appropriately ordered 200 times (16.9%) and inappropriately ordered 984 times (83.1%). Of the 1,437 anti-HBc IgM tests, 1,427 tests (99.3%) were nonreactive, 7 tests (0.5%) were reactive, and 3 tests (0.2%) were indeterminate. Of the 7 reactive tests, 6 were performed appropriately and 1 inappropriately. Of the 3 indeterminate tests, 1 was performed appropriately and 2 inappropriately.

Based on these results, the 2-step advisory screen achieved a statistically significant (*P*<0.0001) but relatively small 10.6 percentage point reduction in inappropriate anti-HAV IgM and anti-HBc IgM testing compared to the preintervention percentages.

In addition, we observed reductions of 22.5% and 21.5% of the overall anti-HAV IgM and anti-HBc IgM antibody testing, respectively, when comparing the total number of tests after implementation of the second intervention with the total number of tests after implementation of the first intervention.

Anti-HCV was tested 4,945 times after the second intervention, and tests were positive 571 times (11.5%). Inappropriate anti-HCV testing was performed 158 times, comprising 3.2% of the total anti-HCV tests. The difference between the preintervention and postintervention inappropriate testing rates for anti-HCV was not statistically significant (*P*=0.34).

Anti-HAV testing was performed 993 times and was positive 516 times (52.0%). Inappropriate HAV total testing in patients with a known positive anti-HAV was performed 13 times, comprising 1.3% of the total anti-HAV tests. As with the anti-HAV IgM and anti-HBc IgM testing, we observed a statistically significant reduction in the postintervention inappropriate testing rate (*P*<0.0001).

Anti-HBc total testing was performed 2,273 times after the second intervention and was positive 296 times (13.0%). Inappropriate anti-HBc total antibody testing in patients with a known prior positive anti-HBc total antibody result was performed 29 times, comprising 1.3% of the total anti-HBc total tests. The difference between the preintervention and postintervention inappropriate testing rates for anti-HBc was not statistically significant (*P*=0.72).

## DISCUSSION

Laboratory testing is an integral part of modern medicine. As with all medical tests, the interpretation of liver chemistries must be performed within the context of the patient's medical history, disease risk factors, symptoms, physical examination, laboratory findings, and established clinical guidelines. For example, when determining appropriate viral hepatitis serologic testing, the magnitude of elevations in AST and ALT should be considered. As both aminotransferases are highly concentrated in the liver, injury to the liver, whether acute or chronic, characteristically increases serum concentrations of aminotransferases. However, no uniform definitions exist for the classification of aminotransferase elevations, as different reviews use different cutoff points.^4-7^ Patients with marked increases in aminotransferase levels (>10 times the upper reference limit) typically have an acute liver injury. However, data from a series of patients with acute liver injury as a result of viral hepatitis suggest that the most sensitive and specific aminotransferase threshold level to identify acute injury lies within the moderate range of increase (5 to 10 times the upper reference limit, at 200 U/L for AST [sensitivity 91%, specificity 95%] and 300 U/L for ALT [sensitivity 96%, specificity 94%]).^8^

Although the appropriate upper reference range for ALT may be 19 U/L in females and 30 U/L in males,^9^ the upper limits of normal for ALT and AST used by the hospital laboratory for this study were 55 U/L and 34 U/L, respectively, and a cutoff of 250 U/L (almost 5 times the upper limits of normal) for either ALT or AST was chosen as the lowest level appropriate for ordering the acute hepatitis serologies (anti-HAV IgM and anti-HBc IgM) for this analysis. A noteworthy observation is that of the 12 reactive anti-HAV IgM tests after the first intervention, 6 were interpreted as inappropriate using the criteria defined by our study (concomitant ALT or AST <250 U/L). Because our data were deidentified, the setting and further clinical characteristics of these reactive tests were unavailable for evaluation. These tests may have been false positives, or the aminotransferase cutoff level we chose for our study may be flawed.

The ramifications of ordering medical testing inappropriately can be both financial and clinical. The financial consequences of inappropriately ordered hepatitis serologies were demonstrated in a study from Dokuz Eylül University Hospital, Turkey, undertaken from May 2002 to May 2005.^10^ Ozbek et al retrospectively evaluated the rate of unnecessary testing of anti-HAV total and anti-HBc total used in serologic diagnosis of hepatitis A and B infections. They demonstrated that 14% of anti-HAV total tests and 18% of anti-HBc total tests were unnecessarily repeated and estimated the cost of the 2,101 inappropriate tests to be $17,000 (USD), an amount that would be substantially more when adjusted for the costs of testing in the United States.^10^ Another Turkish study from Tavşanlı General Hospital demonstrated that in a 2-year period, 1,452 anti-HAV IgM tests, 1,452 anti-HAV total tests, 208 anti-HBs tests, 208 anti-HBc total tests, 1,210 anti-HBc IgM tests, 1,358 HBeAg tests, and 1,216 anti-HBe tests were requested inappropriately.^7^ To our knowledge, no similar US studies investigating the impact of inappropriate hepatitis testing have been published.

Beyond the financial costs, the clinical consequences of ordering inappropriate medical testing are also noteworthy. For instance, reordering screening tests such as hepatitis C antibody in patients known to have been cured of chronic hepatitis C infection can lead to patient confusion, dissatisfaction, and unnecessary referrals to specialists.

Our study demonstrated that at a tertiary care university hospital, inappropriate ordering of acute viral hepatitis serologies (anti-HAV IgM and anti-HBc IgM) was common, likely related to the incorporation of the acute viral serologies in the “hepatitis panel” without calling the panel an “acute hepatitis panel.” Despite 2 interventions resulting in statistically significant reductions in the rates of inappropriate testing, the rates of testing remained high and similar, whether the provider had to justify overriding the automatic alert or not. However, the total number of tests for anti-HAV IgM and anti-HBc IgM was reduced by 22.5% and 21.5%, respectively, after the second intervention when compared to the total number of tests after the first intervention, suggesting that the first intervention not only reduced the rate of inappropriate testing but may have also played a role in educating providers. On the other hand, the rate of inappropriate serology testing for chronic hepatitis is low, and our 2-step intervention only reduced the testing rates for the anti-HAV total tests in this population.

Overall, these findings may indicate that an electronic intervention without additional education may not be enough, and other interventions may be necessary to reduce the rates of inappropriate testing further. Such interventions could include identifying and targeting provider groups that are more likely to order unnecessary tests, avoiding the use of order sets or order panels, developing laboratory utilization committees, and providing other methods of education.

A limitation of this study is that it was conducted at a tertiary single-center care hospital with a major liver treatment center where a high number of patients with elevated liver enzymes are seen. Further, this study was conducted at a teaching hospital where a variety of providers with different education and experience levels order testing. Finally, the interventions used in this study require an EHR that can be modified to include triggered warning screens/alerts.

Results from studies such as ours can be translated into real-life solutions as demonstrated with other studies. For example, in the early 1990s, Bareford and Hayling demonstrated that monthly seminars outlining the appropriate use of laboratory tests reduced unnecessary tests by more than 25% in 6 months.^11^ Sharma and Salzmann demonstrated that including warnings or reminders in electronic ordering systems when unnecessary tests were ordered reduced unnecessary testing by half.^12^

## CONCLUSION

Most of the serology tests for acute viral hepatitis at a tertiary university hospital in the United States were unnecessary. Implementing a simple electronic reminder was associated with a statistically significant 10% reduction of unnecessary tests. The intervention was feasible and implemented at minimal cost. Nonetheless, additional interventions will likely be required to reduce unnecessary testing further.

## Notes

This article meets the Accreditation Council for Graduate Medical Education and the American Board of Medical Specialties Maintenance of Certification competencies for Patient Care, Medical Knowledge, and Practice-Based Learning and Improvement.
