# ERRATUM

**DOI:** 10.31486/toj.21.5018

**Published:** 2021

Hammami MB, Hussaini K, Chhaparia A, Khalid H, Chamberland R, Neuschwander-Tetri B. Inappropriate testing for acute viral hepatitis is common-impact of an intervention using the electronic health record in a tertiary teaching hospital in the United States. *Ochsner J*. 2020 Fall;20(3):293-298. doi: 10.31486/toj.19.0062. PMID: 33071662; PMCID: PMC7529134.

Erratum submitted by the authors. The authors wish to clarify for readers that the quality improvement component of their article did not need and did not have institutional review board (IRB) approval:

The authors apologize that the text of our paper indicated that the implementation of warning messages in our electronic health record (EHR) that would appear during test ordering was identified as research. The implementation of the EHR warnings was a quality improvement initiative rather than research, and this component of the work was not reviewed by the Saint Louis University IRB nor did it need to be reviewed by the IRB. The original data collection from patient records and the analysis of that data was IRB approved.

